# ABCs of Pain: A Functional Scale Measuring Perioperative Pain in Total Hip Arthroplasty Patients

**DOI:** 10.5435/JAAOSGlobal-D-21-00097

**Published:** 2021-06-02

**Authors:** Anneliese N. Hierl, Hannah Kang Moran, Mark R. Villwock, Kimberly J. Templeton, Jennifer A. Villwock

**Affiliations:** From the University of Kansas Medical Center, Otolaryngology—Head and Neck Surgery, Kansas City, KS (Villwock, and Dr. Villwock); the University of Kansas Medical Center, Orthopedic Surgery, Kansas City, KS (Dr. Templeton); and the University of Kansas School of Medicine, Kansas City, KS (Ms. Hierl and Ms. Moran).

## Abstract

Functional pain assessments are critical in total hip arthroplasty. This pilot study investigated a novel functional pain scale—the Activity-Based Checks of Pain (ABCs)—and its correlations with the 0 to 10 numeric rating scale (NRS) and outpatient milligram of morphine equivalents (MMEs) prescribed and needed in the first 2 weeks after total hip arthroplasty.

**Methods:**

ABCs and NRS were collected at the baseline, inpatient, and 2-week follow-up. Primary outcome metrics were needed for pain medication at the time of pain scale completion, MMEs prescribed at discharge, and MMEs taken. Individual ABC functions and composite score were analyzed using Spearman rho and Mann-Whitney *U* tests

**Results:**

ABC and NRS scores were greatest preoperatively (n = 39). At each stage, the ABCs correlated with the NRS (ρ = 0.450, *P* < 0.01; ρ = 0.402, *P* < 0.05; and ρ = 0.563, *P* < 0.01). ABC or NRS scores did not correlate with MMEs prescribed. Last in-house NRS correlated with MMEs taken postoperatively (r = 0.571, *P* < 0.01). Specific ABCs functions—“sitting up” (ρ = 0.418, *P* < 0.01), “walking in room” (ρ = 0.353, *P* < 0.05), and “walking outside room” (ρ = 0.362, *P* < 0.05)—on the day of discharge correlated with MMEs taken.

**Conclusion:**

ABCs scale correlates with NRS. Neither scale correlated with MMEs prescribed at discharge, suggesting pain is undervalued in analgesic planning. Clinicians should assess pain with functions found to correlate with MMEs taken—“sitting up,” “walking in room,” and “walking outside room.”

Although often considered pathologic, pain can also be part of the normal healing process, particularly postoperatively. It is important for pain to be adequately controlled to prevent both unnecessary suffering and complications. In 2000, the Joint Commission on Accreditation of Healthcare Organizations made recommendations that claimed pain was a patients' rights issue and emphasized the need to quantify pain by placing it on a 10-point numeric rating scale (NRS).^1,2^ The validity and safety of this approach was not investigated before its implementation. Pain management difficulties are compounded by the current methods of pain assessment. Noteworthy is that pain is inherently subjective and influenced by an individual's experiences, expectations, and culture.^3^ Yet the complex nature of pain is often reduced to a unidimensional articulation of its intensity, as indicated by the prevalence of the NRS in clinical practice. Although these rating scales are simple and easy to complete, they cannot assess metrics of critical clinical importance and importance to patients, such as the effect of pain on the ability to perform activities of daily life. Furthermore, in 2018, the JHACO released expanded guidelines on pain management to include an emphasis on assessing and managing functional pain.^4^ However, the NRS remains the predominant pain assessment in clinical settings.^5,6^ In the years since, issues associated with analgesic overuse have come to dominate the pain dialog.

In response to the recognized need for a functional pain scale, the Activity-Based Checks of Pain—Functional Pain Scale (ABCs) was developed. Briefly, the ABCs is a novel pain assessment tool that is intended to efficiently, visually, and meaningfully reflect both pain and its functional implications. The purpose of this prospective cohort study was to investigate the correlation of the composite ABCs and the NRS with (1) each other, (2) reported need for pain medication at the time the pain scale was completed, (3) milligrams of morphine equivalents (MMEs) prescribed on postoperative discharge, and (4) patient-reported use of MMEs during the first 2 postoperative weeks in patients undergoing total hip arthroplasty (THA). Secondary outcomes include determining whether there are specific functions within the ABCs are driving the overall pain score and are most predictive of analgesic needs. The main intention of this pilot study was to gain knowledge that could justify a larger, more expansive study to confirm the validity and usability of the functional pain assessment tool developed.

## Methods

### Overview

**Figure 1:**
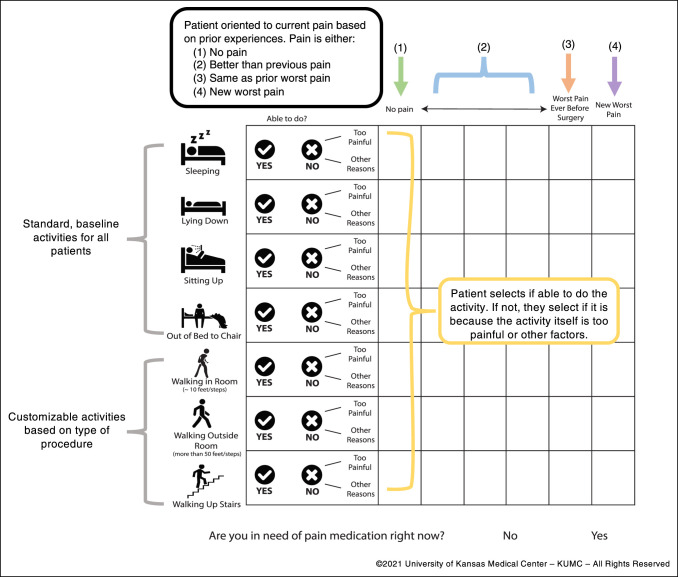
Illustration showing annotated version of Activity-Based Checks of pain scale

This study underwent Institutional Review Board review and received approval before commencement of any study activities. The ABCs' simple and visual format (Figure 1) is intentional because several studies emphasize the importance of infographics in health communication.^7,8,9,10^ Its face validity was established through discussions and informal focus groups with surgeons from multiple subspecialties and key stakeholders in nursing, psychology/psychiatry, and physical therapy. Functions increase in difficulty because the scale progresses from the top to the bottom, asking patients first about pain associated with “sleeping” and progressing to assessing pain with a more involved function such as “walking up a step.”

### Setting and Study Population

This study was done at an urban, academic hospital. The subjects considered for inclusion were English-speaking, older than 18 years, and scheduled to undergo unilateral primary THA. Exclusion criteria included the following: known pain disorders, history of pain medication abuse, revision arthroplasty, traumatic hip fracture, and patients with cognitive disabilities.

### Data Collection

Subjects undergoing unilateral THA were prospectively enrolled the morning of their surgery in the preoperative clinical area. At this time, a study team member oriented the patient to the ABCs and how to correctly complete it.

Subject demographics and SF-12 health scores were recorded. The SF-12 was collected because baseline emotional and physical well-being affect pain. Baseline pain data were collected using the ABCs and NRS. Both scales were completed at the same time. During each subject's inpatient stay, the ABCs and NRS data were collected twice daily as was patient-reported need for pain medication at the time of pain scale completion. For data analysis, each of the seven functions on the ABC scale was assigned a different weight depending on physician perceived difficulty. These values were summed to represent the composite 10-point ABC score. Both the function-specific score and composite ABC score were used in data analysis. Quantity of opioid analgesics prescribed at discharge, in MMEs, was collected. Two weeks after the participants’ surgery date, subjects were contacted through telephone and were asked to report the number of opioids taken and to rate their current pain on both the ABCs and the NRS. If a participant was unable to be reached on the date exactly 2 weeks postoperation, then they were called for up to 3 days after this to gather ABCs and NRS scores and MMEs taken to be included in analysis. Patients with incomplete follow-up data after this 14 to 17 day postoperative period were excluded from analysis.

### Statistical Analysis

Data were analyzed using SPSS version 26. Descriptive statistics for scale variables were reported using the median and interquartile range (IQR). The Spearman rho test was used to assess correlation between scale variables. Group comparisons were done using the Mann-Whitney *U* test. Significance was set a priori at *P* < 0.05.

## Results

Fifty-one patients were enrolled in this study. Nine patients were lost to follow-up after 5 unsuccessful attempts to contact them through telephone postoperatively. Three patients were lost to follow-up after suffering another injury that caused notable pain and a decrease in function that disqualified them from continued study participation. No patients withdrew. Thirty-nine participants completed the study through the 2-week follow-up. The median age of the participants was 59 years (IQR: 51 to 70). Of the 39 participants who completed their 2-week follow-up, 21 were women (53.8%). Five patients were identified as Black (12.8%) and 33 as White (84.6%). State demographics are reflected in our study population with 50.2% woman, 86.3% White, and 6.1% Black.^11^ SF-12 was collected at baseline, and the median score was 27 ± 7.1. SF-12 scores and correlation with composite ABC, NRS, MMEs prescribed, and MMEs taken are given in Table 1. The mental SF-12 significantly correlated with the baseline composite ABCs (ρ = −0.483 *P* < 0.01) and baseline NRS (ρ = −0.388 *P* < 0.05). It did not correlate with MMEs prescribed or taken. The physical SF-12 did not significantly correlate with the baseline composite ABCs, baseline NRS, or MMEs prescribed and MMEs taken. Worse scores on the SF-12 indicate worse mental health. Although higher scores were associated with higher scores on the pain scales, they did not correlate with increased MMEs taken postoperatively.

**Table 1:** Baseline SF-12 Scores Correlated With Composite ABCs, NRS, MMEs Prescribed, and MMEs Taken

	Mental SF 12	Physical SF 12
Baseline composite ABCs	−0.483^b^	−0.255
Baseline NRS	−0.388^a^	−0.300
MMEs prescribed	−0.017	−0.147
MMEs taken	−0.141	−0.194

ABCs, Activity-Based Checks of Pain, MME = milligram of morphine equivalent, NRS, numeric rating scale

a Correlation is significant at the 0.05 level (2-tailed)

b Correlation is significant at the 0.01 level (2-tailed)

Pain on both the NRS and the composite ABCs was greatest preoperatively. The median values throughout the study are given in Table 2. At each stage of the study—baseline, before discharge, and at the 2-week follow-up—the composite ABCs correlated with the NRS (ρ = 0.450 *P* < 0.01; ρ = 0.402 *P* < 0.05; and ρ = 0.563 *P* < 0.01, respectively).

**Table 2:** ABCs and NRS at Each Time-Point of the Study

	Composite ABCs	NRS
Baseline preoperatively	3.7 (2.3 to 5.0)	6 (4 to 8)
Before discharge	2.3 (1.8 to 3.2)	4 (2 to 6)
Two-week follow-up	2.1 (1.7 to 2.6)	3 (2 to 5)

ABC = Activity-Based Checks of Pain, NRS, numeric rating scale

Median (interquartile range)

**Figure 2:**
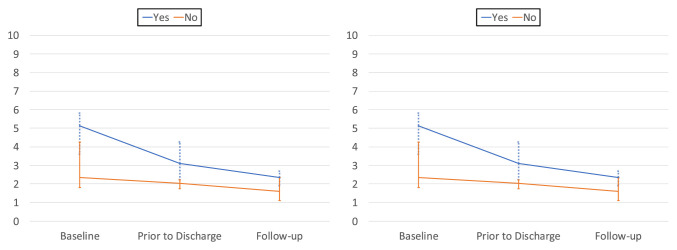
Line graph showing (**A**) median composite Activity-Based Checks (ABCs) of pain based on patient's self-reported need for pain medication and (**B**) median numerical rating scale (NRS) of pain based on patient's self-reported need for pain medication. Error bars indicate interquartile range.

The median NRS and composite ABCs at each stage of this study were then stratified by the patient's self-reported need for pain medication (Figure 2). Pain was always significantly higher in the “yes” cohort when asked whether they were in need of pain medication (*P* < 0.05) with the exception of the NRS at the follow-up, where no significant difference was observed (*P* = 0.429). At baseline, no individual function significantly correlated with “yes” or “no” self-reported need for pain medication. At the last inpatient pain recording, “sitting up” (*P* = 0.046), “walking in room” (*P* = 0.003), and “walking outside the room” (*P* = 0.019) significantly correlated with answering “yes” in need of pain medication. At the 2-week follow-up, “walking up stairs” significantly correlated with “yes” in need of pain medication (*P* = 0.009). A summary of “yes” or “no” self-reported need for pain medication with respect to ABCs, NRS, and the individual functions pain scores is given in Table 3.

**Table 3:** “Yes” or “No” Self-Reported Need for Pain Medication About Composite ABCs, NRS, and the Individual Functions Mean Pain Scores

Time of Collection	Yes	No	*P*
Baseline			
Composite ABCs	4.89	4.47	0.008
NRS	7.30	2.90	0.12
Sleeping	1.88	1.47	0.621
Lying down	2.43	1.50	0.085
Sitting up	1.50	1.05	0.286
Out of bed to chair	2.67	1.95	0.188
Walking in room (>10 ft)	3.00	2.11	0.085
Walking outside room (>50 ft)	3.17	2.50	0.199
Walking up stairs	3.29	2.65	0.187
Last inpatient			
Composite ABCs	3.17	2.08	0.004
NRS	5.64	3.40	0.001
Sleeping	1.17	0.86	0.347
Lying down	1.36	0.87	0.310
Sitting up	1.57	1.00	0.046
Out of bed to chair	2.43	2.07	0.270
Walking in room (>10 ft)	2.46	1.47	0.003
Walking outside room (>50 ft)	2.33	1.46	0.019
Walking up stairs	2.25	1.80	0.360
Two-week postoperative			
Composite ABCs	2.46	1.71	0.040
NRS	4.21	3.20	0.429
Sleeping	1.28	1.10	0.869
Lying down	1.26	1.00	0.456
Sitting up	1.21	0.80	0.138
Out of bed to chair	1.79	1.40	0.286
Walking in room (>10 ft)	1.47	1.00	0.211
Walking outside room (>50 ft)	1.60	1.00	0.149
Walking up stairs	2.29	1.10	0.009

ABC = Activity-Based Checks of Pain, NRS = numeric rating scale

p < 0.05

No notable correlation was observed between composite ABC scores collected at baseline, or before discharge, or at the 2-week postoperative follow-up and MMEs prescribed at discharge. No notable correlation was observed between NRS scores collected at baseline, or before discharge, or at the 2-week postoperative follow-up and MMEs prescribed at discharge. The median MMEs prescribed were 625 (IQR: 450 to 938), with the median MMEs taken being 270 (IQR: 113 to 483). Sex differences in MMEs prescribing and consumption patterns were noted. Men were prescribed less MMEs (median 500, IQR: 450 to 938, *P* = 0.245) compared with women (median 700, IQR: 488 to 938, *P* = 0.245). Although there was no statistically significant difference between sexes in MMEs prescribed, women consumed a smaller proportion of prescribed MMEs than men (42.0% versus 59.2%, *P* = 0.043).

The NRS collected before discharge correlated with MMEs taken (ρ = 0.571, *P* < 0.01) during the 2-week postoperative period. Although the composite ABCs scores did not correlate with MMEs taken in the 2-week postoperative period, pain with 3 functions before discharge were significantly correlated. At the last inpatient scale completion, the following functions—“sitting up” (ρ = 0.418 *P* < 0.01), “walking in room” (ρ = 0.353 *P* < 0.05), and “walking outside room” (ρ = 0.362 *P* < 0.05)—correlated with MMEs taken during the 2-week postoperative period. Finally, although the 2-week NRS did not correlate with the ABCs function, “out of bed to chair” significantly correlated with MMEs taken (ρ = 0.406 *P* < 0.05) during the 2-week postoperative period (Table 4).

**Table 4:** Functions Correlated With MMEs Taken During 2-Week Postoperative Period

Time of ABCs Collection	Functions	Correlation Coefficient to MMEs Taken
Baseline	Composite ABCs	−0.053
	Sleeping	0.038
	Lying down	−0.067
	Sitting up	0.052
	Out of bed to chair	0.072
	Walking in room	0.111
	Walking outside room	0.225
	Walking up stairs	0.214
Last inpatient	Composite ABCs	0.256
	Sleeping	0.327
	Lying down	0.014
	Sitting up	0.418^b^
	Out of bed to chair	0.080
	Walking in room	0.353^a^
	Walking outside room	0.362^a^
	Walking up stairs	0.213
Two-week postoperative	Composite ABCs	0.064
	Sleeping	0.304
	Lying down	−0.162
	Sitting up	0.161
	Out of bed to chair	0.406^a^
	Walking in room	0.111
	Walking outside room	−0.054
	Walking up stairs	−0.123

ABC = Activity-Based Checks of Pain, MME, milligram of morphine equivalent

a Correlation is significant at the 0.05 level (2-tailed)

b Correlation is significant at the 0.01 level (2-tailed)

## Discussion

This study aimed to compare the ABCs—a simple, visual assessment of postoperative pain and its effect on function—with the NRS about four outcomes as follows: (1) ABCs and NRS to each other, (2) reported need for pain medication at the time the pain scale was completed, (3) milligrams of morphine equivalents (MMEs) prescribed on postoperative discharge, and (4) patient-reported use of MMEs during the first 2 postoperative weeks in patients undergoing THA. This is a pilot study that was done to gain knowledge to justify a larger, more expansive study to confirm the validity and usability of the functional pain assessment tool developed. We found that pain levels with three ABCs functions—sitting up, walking in room, and walking outside of the room—on the day of discharge markedly correlated with self-reported MME needs in the first 2 weeks after surgery. The composite ABCs score did not correlate, indicating that pain with certain functions may be more important than global ratings of pain for analgesia needs. Interestingly, neither the NRS nor the composite ABCs score correlated with MMEs prescribed at discharge, indicating that prescribing is standard for a given procedure, rather than taking into account the patients’ reported pain and function. Although some individual functions did correlate with MME needs, the composite ABCs did not. This illustrates that although the functional recovery in the perioperative period from THA is dynamic and potentially difficult to adequately assess, there are functional markers than can guide management postoperatively. The ABCs composite score of all functions correlated with the NRS at baseline, throughout the hospital stay, and at the follow-up. The NRS at discharge was correlated with MMEs used. However, patient reported the need for medication at the time of pain scale completion was even more strongly correlated. This suggests that unidimensional measurements of pain intensity may not be useful for guiding analgesia prescribing decisions, highlighting one of the benefits of linking pain with function during assessments. This may indicate that patients believe pain is pathologic and requires treatment, rather than basing decisions about analgesia on how pain is affecting their functionality. Although not a primary outcome, we also noted significant differences in pain prescribing and usage patterns between men and women patients.

Pain associated with performance of functional tasks is important to assess, especially in the postoperative setting when patients must meet various functional milestones to be discharged.^12^ Common hospital discharge criteria include the ability to independently transfer in and out of bed, a chair, and a toilet seat; independently ambulate approximately 150 feet; and independently negotiate stairs.^13^ Previously developed pain assessment studies have attempted to include functional metrics. For example, the Osteoarthritis-Function-Computer Adaptive Test (OA-FUNCTION-CAT) was designed for clinical research purposes to track outcomes in patients with osteoarthritis. The test consists of 125 written prompts regarding functional activities. The patient assesses a range of functions—“turning in bed” to “sitting down on a soft, low couch” to “jumping and landing on your right leg.”^14^ Such large numbers of functions to assess, however, can lead to survey fatigue and overall difficulty completing assessments. The Defense and Veterans Pain Rating Scale, which is similar to a NRS, also attempts to assess function, containing a question on sleep and activity.^15^ The Defense and Veterans Pain Rating Scale reportedly included these functions to urge the patient to consider how pain affects their life and provide the opportunity to gauge longitudinal progress.^16^ Although these potentially represent improvements over unidimensional scales, they have not been implemented to guide real-time decision-making.

The ABCs scale is unique in multiple ways. First, it uses infographics illustrating the activity in question, whereas most other pain assessment tools only contain text. Infographics are an emerging tool used in health care to enhance the understanding of health conditions and give patients the ability to make decisions for themselves.^17^ Language barriers, time constraints, and disparities of the education level may be mitigated by tools that bridge communication gaps, such as infographics.^18^ In addition, the ABCs assesses functions that can be done both in a hospital and at home. Patients complete the same checklist hours after their surgery as they do 2 weeks after the surgery. This allows for consistent longitudinal pain and function monitoring. Although not investigated in this study, the ABCs was designed to be customizable to reflect the surgery done and anticipated pain associated with that specific surgery that may limit function. This would allow surgeons and other providers to add additional functional outcomes specific to their patient population.

The scale used in this study contained important functions to assess, in increasing order of difficulty, as determined by several key informant interviews with orthopaedic surgeons. Interestingly, we found that “walking up stairs,” which was deemed by the surgeons to be the most difficult postoperative task and therefore placed at the bottom of the scale, was typically not the most painful for our participants at different parts of their recovery. However, this study showed that sitting up, walking in room, and walking outside room were the most important functions before discharge, and out of bed to chair was the most important function at the 2-week postoperative follow-up. Functional importance was determined because these are the actions that markedly correlated with MMEs taken. More studies of pain related to function may help determine which functions are the most important and predictive of analgesic needs from the patient perspective, rather than the provider perspective. This insight is important to facilitate meaningful communication between patients and their care teams. For example, previous studies have shown that patients report higher self-efficacy, lower pain intensity, and less pain interference within their lives if they perceive patient-centered communication.^19^ However, many patients still feel as if their pain is not understood and undertreated even when receiving opioid prescriptions.^20^

At discharge, patients were not consistently prescribed the same pain control regimen within our cohort. The range of MMEs prescribed was 450 to 939 for men and 488 to 938 for women. The reasons for these variations are unclear, given that the composite ABCs, individual functions included on the ABCs, or NRS correlated with MMEs prescribed at discharge. Furthermore, all patients in our study underwent analogous procedures. Complex, bilateral, and traumatic cases were intentionally excluded. Noteworthy is that procedure-specific opioid prescription guidelines are available, but they are often based on expert opinion rather than evidence and tend to be quite broad.^21^ For example, the Michigan OPEN recommendations for the quantity of 5 mg hydrocodone-acetaminophen tablets to prescribe after THA are 0 to 30 tablets (0 to 150 MMEs).^22^ In our study, subjects consistently received opioid prescriptions that ranged from 3 to 6 times this amount. High-risk prescribing is determined to be greater than 90 MMEs per day and therefore suggests a higher risk of adverse events.^23^ According to this designation, all of the participants in our study were receiving high-risk prescriptions and were thus more susceptible to adverse events.

Procedure-specific, restrictive opioid protocols are gaining popularity, likely to ensure similar treatment for similar pathologies. However, previous studies have cautioned against using unidimensional patient-reported rating of pain to decide opioid prescription choices because these pain scores fail to account for the subjective, complex nature of pain and because their use has been implicated in fostering the prescription opioid epidemic.^24-26^ Our findings similarly support the importance of multimodal and functional pain assessment. For example, we found 3 functions on the day of discharge—“sitting up,” “walking in room,” and “walking outside room”—to correlate with MMEs needed in the first 2 weeks after postoperative discharge home. These individual functions were more important than the composite ABCs score. At discharge, the NRS did correlate with MMEs taken at r = 0.571. However, at the 2-week follow-up, only “out of bed to chair” correlated with MMEs taken, indicating that assessing this function at the 2-week follow-up is important for understanding a patient's analgesic needs at that point of dynamic functional improvement. In addition, pain ratings are interpreted differently among clinicians and patients based on their previous experiences with pain, culture, and comorbidities (eg, depression and anxiety), further demonstrating the need for tools to facilitate physician-patient communication regarding pain management plans.^27^ Pain assessment linking pain with function provides a better contextual understanding of pain and may facilitate physician-patient communication regarding pain management plans and expectations. This is particularly important in the setting of procedures, such as THA, which aim to improve function.

In our study, we found that female patients took a smaller proportion of their prescribed MMEs than male patients did at our 2-week follow-up time point. The proportion of MMEs taken as prescribed, 42.0% for women and 59.2% for men, indicates that providers are overprescribing to both sexes, leaving opioids unused, and potentially available for misuse. Although there is evidence that opioid use in women has increased in the past decade, the women in our study still took fewer of their prescribed opioid analgesics than the men.^28,29^ One explanation for this is that female patients with cancer have been found to be more hesitant to take pain medication and adhere to prescribed pain regimens despite reporting greater pain intensity than their male counterparts.^30^ Interestingly, we found no differences in pain reporting between male and female patients, yet the male patients took higher proportions of their prescribed pain medication. Further research is needed to delineate the biological and social underpinning for this phenomenon.

The study was not without limitations. The sample size was relatively small for 39 patients. However, subject demographics closely reflect that of the state in which the study was done, enhancing external validity. Twelve patients were lost to follow-up between discharge and first postoperative visit. Administering the visual survey over the phone could have led to researcher bias because we were the ones interpreting the responses and recording the results. The 2-week follow-up call was done over the phone, and therefore, patients self-reported the number of MMEs taken. We did not collect data on when these pain medications were consumed but rather collected a total number over the 2-week postoperative period. We attempted to minimize the variability in the telephone 2-week postoperative survey delivery by having the same two research team members call the patients and following a predetermined script. The process of self-report is subject to recall bias and social desirability bias. The latter is particularly true for behaviors that may be perceived as high-risk such as taking large amounts of opioid analgesics. However, we do not think that these factors markedly biased results because patients were asked to count remaining opioid medications to determine how many had been taken. Finally, study team members were not involved in the clinical care of subjects, and study results were not shared with their surgical teams. The scale does leave room for interpretation on certain function such as “sleeping” or “lying down.” Anecdotally, some patients did not count “napping” as sleeping, and there was ambiguity about what constitutes “lying down” versus “sitting up.” Study participants were enrolled the day of surgery, and information relayed on the day of surgery may be difficult for patients to retain, given that they typically have to manage a large amount of information from a variety of different providers on the day of their surgery. Surgery under general anesthesia also has the potential to cause retrograde amnesia. However, we do not think that this markedly affected results because research persons were present and available to answer any questions each time the patient completed the ABCs pain scale. Finally, it is important to remember that patient experience of pain is inherently variable and subjective, influenced by precounseling by the surgical team, previous experiences with pain, or cultural norms surrounding expressing pain. Future work will include qualitative components of the patient pain experience to inform future iterations of the ABCs and ensure that it is of maximum benefit to both patients and their care teams for assessment of, and communications about, pain.

## Conclusions

This study demonstrated that pain with specific functions on the ABCs after THA before discharge is correlated with MMEs taken in the first 2 postoperative weeks. Pain levels did not influence the amount of MMEs prescribed at discharge. Assessment tools, such as the ABCs, that include functional metrics may better communicate pain and analgesia needs. Clinicians should incorporate function, especially those found to correlate with MMEs taken—“sitting up,” “walking in room,” and “walking outside room”—and functional improvement postoperatively to guide prescribing patterns. Pain assessment tools, and subsequently patient-provider communication, require functional measures to adequately capture pain management needs. Additional studies are needed to validate this pilot study among a larger, more diverse cohort.
